# A Dynamic Trust-Related Attack Detection Model for IoT Devices and Services Based on the Deep Long Short-Term Memory Technique

**DOI:** 10.3390/s23083814

**Published:** 2023-04-07

**Authors:** Yara Alghofaili, Murad A. Rassam

**Affiliations:** 1 Department of Information Technology, College of Computer, Qassim University, Qassim 51452, Saudi Arabia; 2 Faculty of Engineering and Information Technology, Taiz University, Taiz 6803, Yemen

**Keywords:** Internet of Things, trust management, trust-related attacks, deep learning, long short-term memory

## Abstract

The integration of the cloud and Internet of Things (IoT) technology has resulted in a significant rise in futuristic technology that ensures the long-term development of IoT applications, such as intelligent transportation, smart cities, smart healthcare, and other applications. The explosive growth of these technologies has contributed to a significant rise in threats with catastrophic and severe consequences. These consequences affect IoT adoption for both users and industry owners. Trust-based attacks are the primary selected weapon for malicious purposes in the IoT context, either through leveraging established vulnerabilities to act as trusted devices or by utilizing specific features of emerging technologies (i.e., heterogeneity, dynamic nature, and a large number of linked objects). Consequently, developing more efficient trust management techniques for IoT services has become urgent in this community. Trust management is regarded as a viable solution for IoT trust problems. Such a solution has been used in the last few years to improve security, aid decision-making processes, detect suspicious behavior, isolate suspicious objects, and redirect functionality to trusted zones. However, these solutions remain ineffective when dealing with large amounts of data and constantly changing behaviors. As a result, this paper proposes a dynamic trust-related attack detection model for IoT devices and services based on the deep long short-term memory (LSTM) technique. The proposed model aims to identify the untrusted entities in IoT services and isolate untrusted devices. The effectiveness of the proposed model is evaluated using different data samples with different sizes. The experimental results showed that the proposed model obtained a 99.87% and 99.76% accuracy and F-measure, respectively, in the normal situation, without considering trust-related attacks. Furthermore, the model effectively detected trust-related attacks, achieving a 99.28% and 99.28% accuracy and F-measure, respectively.

## 1. Introduction

**Figure 1:**
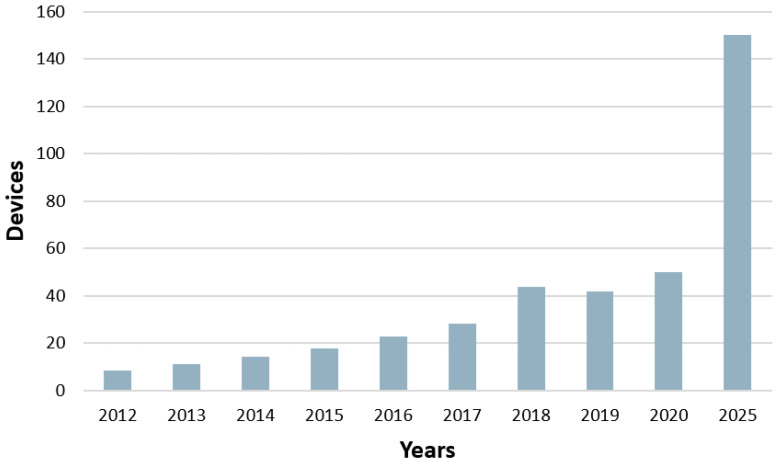
Growth in IoT devices.

The information technology (IT) industry has recently seen rapid growth as it has become an integral part of our daily lives. The Internet of Things (IoT) is a modern technology used in many aspects of life, including agriculture, education, water management, home security, smart grids, and others. As a result, more and more objects are becoming connected daily [1,2,3]. According to [4], the number of objects connected to the IoT reached 50 billion in 2020, and this is expected to increase three-fold by 2025, as shown in Figure 1.

The IoT infrastructure supports several cutting-edge services (IoT services), where many heterogeneous objects collaborate to achieve a shared goal. IoT services have received increasing attention in recent years across various industries. The characteristics of the IoT, such as the diversity of shared data, dynamicity, and device heterogeneity, present entirely new challenges to IoT services and devices. These challenges are addressed mainly by focusing on general security issues rather than assessing the subjective risks associated with IoT entities and service fields [5]. Furthermore, it might result in catastrophic harm and unknown dangers if the information were used for malicious purposes. The trust principle in the IoT can be viewed as a critical feature for establishing trustworthy and reliable service provisions between different objects [6]. As a result, trust is one of the essential requirements for achieving security.

Trust is an amorphous concept with varying definitions depending both on the participants and situations, and it is influenced by measurable and non-measurable variables [6]. This demonstrates that trust is a very complex concept. Other factors contributing to trust include an object’s abilities, strength, reliability, goodness, availability, and other characteristics [7]. As a result, trust management is more complex than security itself, particularly in emerging information technology fields such as the IoT [8].

Trust management is regarded as a viable solution for IoT trust problems. Such solutions have improved security, aided decision-making processes, detected suspicious behavior, isolated suspicious objects, and redirected functionality to trusted zones [9]. Researchers have devised several strategies to address trust concerns, such as those in [10,11,12,13]. These solutions, however, are still unable to fully address trust issues and face several difficulties, including inefficiency in handling large amounts of data and constantly changing behaviors, difficulty in quantifying uncertainty for untrusted behaviors and selecting the best trust model components, and dealing with the heterogeneity and dynamic nature of the IoT while concentrating on a single and unique attack.

This paper proposes a dynamic trust-related attack detection model for IoT devices and services based on the LSTM technique to address the concerns above. This model is capable of detecting untrustworthy behaviors and taking appropriate action. The primary contributions of this paper are (1) creating an intelligent solution based on the LSTM technique that combats the continuing change in behavior and is compatible with big data, and (2) evaluating the model under different conditions (three scenarios of trust-related attacks and different sizes of datasets).

The remainder of this paper is structured as follows: Section 2 presents the background on the concept of trust and its related attacks. Section 3 investigates existing related works to highlight their shortcomings that need to be considered. Section 4 describes the proposed model and the underlying technique used. Section 5 reports on the experimental investigation and model evaluation. Section 6 concludes the paper and suggests some future research directions.

## 2. Trust Concept and Trust-Related Attacks

Trust has been extensively studied in the fields of economics, social sciences, cyberspace, and philosophy [9]. The concept of trust can be seen as complex and needs to be clarified because it depends on the person’s context and view. For instance, the study in [14] defined trust as the desire for the interdependent relationship between the source of trust and the trustee in satisfying the obligations to perform the expectations promised in a particular context, irrespective of the capacity to monitor or control the trustee.

In addition, the study conducted in [15] defined trust in the context of the IoT, such as entity trust, device trust, and data trust, among others, in particular instances where entity trust refers to participants’ expected behavior with respect to aspects such as individual preferences or services. It is also important to acknowledge that device trust could be used for trusted computing and developing computational trust. Moreover, trust data may be extracted via aggregation from untrusted sources or generated from IoT services where trust assessments are necessary [16].

The study conducted in [17] defined trust as a service mechanism that automatically navigates through a wide array of barriers to enable proper decision making based on trust between both parties.

The authors of [18] defined trust as the quality of data generated from IoT systems that flow between sensors and devices.

Another study defined trust as the edge that connects the technological ecosystem with intelligent objects. This definition is based on IoT devices’ capacity to conduct various measurements in smart environments, such as humidity, temperature, and pressure measurements and fire detection, to enable administrator decision making and instant reactions. The suggestions in this study emphasize the need to trust the involved device(s) to properly assess and highlight the relationship between individuals by leveraging trustee feedback to develop appropriate reactionary measures [5].

Based on the previous studies that have defined trust, trust includes multiple concepts such as confidence expectation, dependency, reliability, comfort, vulnerability, context specificity, attitude to risk, utility, and lack of control. This has contributed to the lack of a clear definition of trust. On the other hand, the significant objectives of trust management are, nonetheless, to leverage security, enable decision-making processes, detect untrusted behavior, isolate untrusted entities, and redirect IoT functionality to trustworthy zones.

The explosive development of IoT devices has contributed to a significant rise in threats, since IoT environments integrate many heterogeneous sensors to provide various wide-ranging intelligent services. These sensors may be inaccurate and carry out attacks linked to trust. In contrast, others can enable their allies to collaboratively target a specific service provider to undermine their credibility and improve their allies’ reputations [19]. The most common forms of trust-related attacks are good-mouthing, ballot-stuffing, on–off, bad-mouthing, and discrimination [20]. Table 1 summarizes these attacks.

This paper will cover two types of trust-related attack (bad- and good-mouthing) as part of the experimental evaluation.

## 3. Related Works

Lately, the incorporation of trust management systems has gained significant attention for addressing the issues posed by insecure IoT-based systems and services. Numerous studies have provided models for trust management that can recognize unreliable entities. For example, the study in [24] proposed a trust management protocol involving cooperativeness, honesty, and community interests. By adopting these parameters, it was feasible to generate new networks to build trust connections with existing vertices and survive hostile situations. However, the main drawback of this research is that it employed multimodal logic but did not present a way to aggregate data.

The study in [25] used two models, subject and object, to create a consistent framework based on the robustness of objects. In the initial model, each node continuously adapted its behavior by calculating its connections based on its experiences and the viewpoints shared with providers. In the other approach, the data for each node were allocated and deposited via a distributed hash table structure; hence, any node could consume consistent data. This information was accessible to all nodes but was only processed by specific nodes to prevent exploitation. The experimental evaluation demonstrated the effectiveness of their proposed systems in isolating malicious nodes. Although the model attained a better result, it could have been more effective with newly encountered nodes since maintaining the hash table requires a trustworthy node.

In a later study [10], the authors created a framework of trust propagation for IoT systems. The framework relied on distributed collaborative filtering to generate a response based on social connections, similarity evaluations of connections, and interest links, with the public functioning as the filter. This model evaluated trust convergence, resiliency, and accuracy properties using the NS-3 simulator. The results showed that the model’s efficacy against EigenTrust and PeerTrust was affected by the existence of nodes with malicious intent that execute false recommendations and opportunistic service attacks. These nodes accomplish this by taking advantage of vulnerabilities in the service-oriented architecture (SOA)-based IoT environments. However, the work concentrated only on persistent attacks such as bad-mouthing, opportunistic service, and self-promotion.

In addition, study in ref. [26] developed a straightforward trust management approach based on entropy and Bayesian concepts. The trust value for direct nodes was computed using Bayesian methods and was frequently updated. Likewise, an entropy-based theory assigns weights to different degrees of trust. Thus, the approach can alleviate problems triggered by subjective weight distribution and promote model scalability. In terms of preventing attacks and energy consumption, simulation experiments were presented to determine the efficiency of the recommended trust management approach. The Bayesian model has certain limitations, such as computational cost and the inability to handle multiple nodes.

The authors of [11] proposed a distributed trust management system for the IoT. In a multi-service IoT, the purpose of the system is to identify suspicious node behavior and prevent significant on–off attacks. The model was divided into three phases: neighbor discovery, service request, and trust estimation. The results demonstrated the effectiveness against on–off attacks and a perfect performance in detecting malicious nodes in the network. Despite this, the model is not suitable for large numbers of nodes because, by increasing the nodes, the computation time also increases.

In [13], the authors proposed an IoT trust- and reputation-based technique employing probabilistic neural networks (PNN). The proposed system was tested on IoT edge systems to differentiate between unreliable and reliable nodes. Additionally, the algorithm resolved the issue of initial value trust in the IoT context by predicting rankings for new devices based on their properties and improvement over time. However, this study did not provide any results or recommendations for action.

In another report [27], a central trust management architecture for the IoT was proposed to facilitate sharing of reliable data across IoT systems. The design incorporated a super-node that acted as a centralized trust manager. The super-node saved the trust values of all master and group nodes in a centralized database. In addition, the super-node also tracked other activities, including the network stream and trust administration over all IoT systems. The database operates as a routing table, recording reliable data and the network structure, and manages all sensors in the CTM-IoT platform, further identifying which sensors must join which cluster. Conversely, the centralized model increases the payload for network communication. At the same time, decision making based on passive trust lowers the cluster head election convergence speed.

In [6], the authors introduced an ML concept of computational trust for IoT systems. This model employed two methods: k-means for clustering and labeling tasks, such as recognizing the number of groupings and initial centroid positions, and SVM for classification problems, including recognizing the limits between reliable and unreliable interactions. The model’s effectiveness was proven through a simulation. The results demonstrated that the model achieved 89.74% prediction with a 0.4175% false positive rate. However, using SVM as a classifier is not good because it requires extensive memory and takes a long training time.

The study reported in [28] recommended optimized algorithms for managing IoT trust, considering memory boundary trust value restrictions for all networks. First, the approach proposed a novel clustering method. Second, the method set the conditions over which a network node in IoT trust management can be converted to a particular new master node, the first algorithm having established the conditions. Third, the algorithm was employed to combat bad mouth attacks (BMA). Finally, the algorithm offered techniques through which master nodes can monitor the integrity of cluster nodes and attempt to remove some of them. The empirical outcomes demonstrated the effectiveness of the suggested algorithms.

A further study [29] developed a strategy based on fuzzy logic for identifying on–off attacks, conflicting activity attacks, and other malicious nodes. The proposed method utilized a messaging system related to serial transmission for end-to-end message encryption. The protocol applied fuzzy logic to identify malicious nodes and restrict their untrusted role in generating inaccurate suggestions related to network nodes. The findings proved the approach’s efficacy in various situations, where the average trust value was 65% in both large and small networks. However, fuzzy models depend on human rules and knowledge and are not commonly used because of the acquisition of inaccurate data.

In addition, based on fuzzy logic, the study in [12] established a model that uses criteria, including device proprietorship trust, system security, and security level, to estimate the trust value of a device. Using the user-selected threshold, the fuzzy logic approach is employed to evaluate the level of trust. When the trust level surpasses the threshold, IoT service consumers can also actively choose the entire network entrusted with acquiring their data. The results indicate that IoT nodes are impacted by increasing degrees of trust with greater input attribute values. However, fuzzy logic might not be regarded as a proper solution to creating trust because it depends entirely on human knowledge and experience.

The study in [20] proposed a trust evaluation framework employing a feedforward ANN. The proposed framework enabled the detection and segregation of trust-related attacks initialized by malicious nodes to build a safe environment. The framework was tested based on simulations applied to a publicly available database, Sigcomm. The performance was evaluated using recall, precision, and F-measure, where the results indicated a recall of 94.4%, a precision of 95.68%, and an F-measure of 95.03%. However, it is not clear how the model was trained, as the effectiveness of such models depends on the quality of the training process.

The study in [30] recently developed a neural-network-based trust management approach. This approach considered a variety of viewpoints (such as that of the owner, device, and service). Each view examined certain aspects, including social and location aspects for the user, reputation for the device, and dependability for the product. The approach was evaluated using precision, recall, and F-measure. The research in [31] proposed a flexible trust management technique for wireless sensor networks. Initially, the main integrity of the node is established by analyzing its connection to regional data. Then, the complete trust value is computed utilizing the power assessment and trust recommendation value of other nodes with a high level of trust. Finally, the control of the nodes and their dependability is regularly upgraded. The simulation and analysis findings demonstrated that the trust level of the node determined by this technique wholly and correctly reflected its trustworthiness. However, the dependency on the value of other nodes, which might be malicious, in the trust calculation may give wrong recommendations and therefore wrong overall trust values.

The study in [32] suggested a dynamic trust management mechanism for wireless sensor networks (WSNs). Firstly, the node’s direct trust value is determined by evaluating its performance from interactions with regional information. The total trust value is computed by combining the energy evaluation and trust recommendation values of other nodes with high trust levels. Finally, the node management and node reliability are updated regularly. The simulation and analysis results demonstrated that the node’s trust level determined by this technique wholly and accurately reflected its trustworthiness. However, relying on the values of other nodes, which may be malicious, in trust calculations may result in incorrect recommendations and abnormal overall trust values.

Another study [33] proposed an information entropy trust analysis strategy to address the trust problem in the IoT communications terminal power distribution. The proposed strategy was used to determine the direct trust value created based on the significance of an exponential distribution; subsequently, the selected forgetting factor and sliding windows were utilized to adjust the direct trust value. In addition, the unpredictability in the direct trust value was tested. The indirect trust value was added to account for direct trust judgment errors. Furthermore, indirect and direct trust values were systematically analyzed to increase the precision of assessments. The experimentation demonstrated that the technique could successfully resist collision and resist attacks. However, the model has many drawbacks in determining the weight, as many parameters and complex calculations are involved.

In [34], a model-based, unified fuzzy-based computational technology and a multiple-criteria decision-making approach were suggested. The trust weights were assessed utilizing this model. The research demonstrated that the fuzzy mechanism in multiple-criteria decision-making performed admirably in selecting trustworthy friends in a SIoT environment and could be utilized as a crucial tool for the social objects in SIoT to uncover trust-building characteristics.

Recent research [35] presented a framework for trust management in Internet of Things devices and products created by the simple multi-attribute rating technique (SMART) and the LSTM algorithm. The SMART approach was utilized to calculate the trust value. At the same time, the LSTM method was employed to recognize behavior changes based on the trust threshold. Different performance metrics were utilized, such as precision, recall, and F1-score, to test the performance of the proposed system. The proposed framework achieved 99.87% accuracy and 99.76% F-score. However, the study did not cover trust-related attacks.

Table 2 summarizes how the current trust management techniques deal with trust-related attacks.

To conclude, managing trust is an essential issue perceived as a big challenge for the IoT industry. As has already been covered in this section, several techniques have been offered to manage trust in the literature. However, the following list of significant research gaps still needs to be filled in:

(1) Dealing with Trust-related Attacks

Attackers influence the behavior of untrusted entities while posing as trustworthy entities; identifying these misbehaviors is crucial. Existing research either ignores or only focuses on a specific type of attack, as shown in Table 2, yet an attacker may opt for a complex approach to behave malevolently.

(2) Shortcomings of Techniques Used

Most of the present research and advancements in trust management has centered on implementing statistical methods or ML approaches, such as those reported in [29]. These strategies have several disadvantages, including their lack of effectiveness when addressing the massive volume of data and continually varying behavior, their high memory consumption, and their inability to measure the risk of untrusted activities. Therefore, deep learning strategies may be an excellent alternative for addressing the limits of machine learning and statistical methods. Deep learning is widely employed in various applications, including computer vision, NLP, robotics, and misbehavior detection [36]. Deep learning has many pros over ML and statistical techniques, such as the following: (1) owing to the use of different hidden layers within a neural network topology, deep learning can match complex nonlinear networks between parameters [36]; (2) it is particularly well suited to address “big data” issues [36]; and (3) it can train IoT devices with more sophisticated behavioral patterns than ML and statistical methods [36].

### Concept of our Solution

Trust issues can contribute to a lack of willingness to use IoT services or devices. According to the research and development works that were reviewed and critically discussed in the previous section, trust has many issues related to trust behaviors between entities, the limitations in current techniques, and the IoT heterogeneous environment. The following points detail the issues: (1) For trust behaviors, the untrusted behaviors are continually changing and the suspicious behaviors become hard to detect because the tools used by attackers have become more effective. (2) The limitations of current techniques—the existing trust management techniques concentrate on a single and unique attack, but an experienced attacker can choose an intelligent strategy to act maliciously. More significantly, practical intruders can execute multiple attacks simultaneously. Finally, (3) IoT nature—the heterogeneous IoT environment can present issues in handling the quality of the services provided. In contrast, these services come from many resources.

This research offers a solution to the issues discussed in the previous section. The solution is described according to the following steps:

(1) This research employs the deep LSTM technique to address the issues surrounding changes in suspect behavior types and calculate the value of the trust, which can dynamically adapt to new patterns and detect types of untrusted entities that were hitherto unseen. According to Field’s [36] research, the DL technique is based on many hidden layers within the neural network structure, which can fit into dynamic relationships between attributes. Consequently, DL can significantly help users evaluate device and event activity in IoT systems. Moreover, DL could make learning sophisticated behavior patterns easier for IoT devices.
(2) To evaluate the effectiveness of the proposed model in detecting untrustworthy behavior, the model is tested in the presence of one or more trust-related attack scenarios.
(3) To deal with IoT heterogeneous environment issues, this research considers intelligent techniques (i.e., deep LSTM) to deal with the IoT environment and the big data generated. RNNs have been developed to resolve serial data or time series (sensor data) of different lengths/forms to solve behavioral issues. As mentioned in [37], LSTM can effectively tackle IoT issues, especially in detecting behaviors, since it can be correlated with current and previous behaviors. Therefore, this research takes advantage of deep LSTM and tests it under different conditions and with different data sizes.

## 4. The Proposed Model

**Figure 2:**
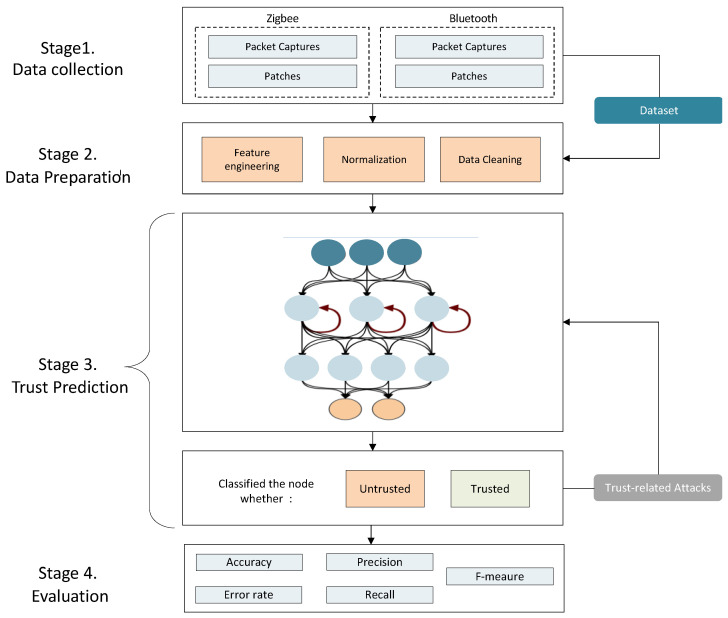
The proposed model.

The proposed model is divided into four main stages: data collection, data preparation, trust prediction, and evaluation, as illustrated in Figure 2.

Stage 1. Data Collection. Data from packet captures and batch patches proposed in [38] were used in this investigation. The data were gathered through smart home activities, which included IP (such as Ethernet, Wi-Fi, and PPP), Bluetooth, Z-Wave, RF869, and ZigBee protocols, within 10 days. Table 3 and Table 4 show details of the devices and the number of captures and patches.
Stage 2. Data Preparation. In this stage, three sub-stages are present: feature engineering, normalization, and data cleaning.

**Table 3:** Device deployment locations [38].

Device Type	Protocol	Placement
Motion sensor	Zigbee	Living room
Motion sensor	Zigbee	Kitchen
Motion sensor	Zigbee	Bathroom
Motion sensor	Zigbee	Bedroom
Door sensor	Zigbee	Entrance door
Door sensor	Zigbee	Dishwasher
Weight scale	Bluetooth	Nearby the gateway
Blood pressure meter	Bluetooth	Nearby the gateway
Gateway	Bluetooth	Office
Gateway	Zigbee	Office
Motion sensor	Zigbee	Living room

**Table 4:** Number of packets and patches for each protocol [38].

Protocol	Packet Captures	Patches
Zigbee	73,876	27,385
Bluetooth	541,544	22,202

**(a)**  **Feature Engineering**

The primary goal of feature engineering is to create new features from existing data [39]. This sub-stage creates new features (e.g., packet loss, delay, and throughput), as achieved in [35].

- Packet loss refers to a packet’s inability to reach its intended target.
- Delay is the lag time that results from transmission from one point to another.
- Throughput refers to the real bandwidth that is measured for the purpose of moving files of a certain size at a specific time and under a specified set of network conditions.
**(b)**  **Normalization**

To produce an accurate result, the data are scaled to values ranging from 0 to 1. This step is required to transform the dataset’s numeric column values so they can be used on a common scale without distorting the variation in value ranges or losing data [40]. Normalization is performed using Equation (1):

(1) $$z_{i}=\frac{x_{i}-\min\left(x\right)}{\max\left(x\right)-\min\left(x\right)\;}$$

where $x_{i}$ is the dataset’s *i*th value, min(*x*) is the dataset’s minimum value, and max(*x*) is the dataset’s maximum value.

**(c)**  **Data Cleaning**

This sub-stage cleans the data by ensuring the validity of dataset samples, such as by removing null and negative values from records.

Stage 3. Trust Prediction. This stage uses the LSTM technique that has recently piqued the scientific community’s interest. LSTM has produced remarkable results when used to solve complex problems such as language translation, text production, and automatic image captioning, among other applications [41]. The method, as seen in [42,43], has been extensively used in recent years to fix security-related concerns. This paper uses LSTM to identify suspicious actions that may indicate problems with trust. The LSTM consists of three gates, as in [44].

**(a)**  **Forget gate**

In the first step of LSTM, the sigmoid cell of the oblivion gate determines what information must be deleted from the LSTM memory. A value of 0 indicates that the item was completely discarded, whereas a value of 1 indicates that it was entirely retained [41]. The value is calculated using Equation (2):

(2) $$f_{t}=\sigma \left(Wf\;•\;\left[h_{t-1},\;x_{t}\right]+b_{f}\right)\;$$

where $b_{f}\;$ is a constant referred to as the bias value.

**(b)**  **Input gate**

The second step of the LSTM is to use the input gate to determine what information to store in the LSTM memory based on the cell state [41]. The values of the input gate are calculated using Equations (3)–(5):

(3) $$i_{t}=\sigma \left(W_{i}\;•\;\left[h_{t-1},\;x_{t}\;\right]+b_{i}\right)$$

(4) $${\bar{c}}_{t}=tanh\left(W_{c}\;•\;\left[h_{t-1},\;x_{t}\right]\;\right)+b_{c})$$

(5) $$C_{t}=f_{t}\;*\;C_{t-1}+i_{t}\;*\;{\bar{c}}_{t}$$

where $i_{t}$ specifies whether or not the value should be updated, $C_{t}$ represents a new vector of candidate values, $\bar{C_{t}}$ is the cell information, and $f_{t}$ is the forget gate parameter with a value between 0 and 1, with 0 indicating complete removal and 1 indicating complete retention.

**(c)**  **Output gate**

**Figure 3:**
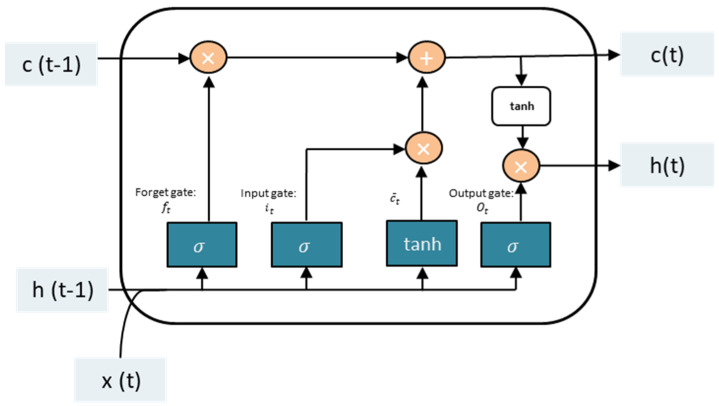
LSTM single cell.

Following cell state updates, it is necessary to determine which output cell state attributes depend on the inputs $h_{t-1}$ and $x_{t}$ [41]. Equations (6) and (7) are used in the computation in this gate:

(6) $$O_{t}=\sigma \left(W_{o}\;•\;\left[h_{t-1},\;x_{t}\;\right]+b_{o}\right)$$

(7) $$h_{t}=O_{t}*\;tanh\left(C_{t}\right)$$

where $O_{t}$ denotes the output value and $\;h_{t}$ denotes a number between −1 and 1. This step decides which portion of the cell state will be sent to the next neural network or instant (Figure 3).

Stage 4. Evaluation. In this stage, the proposed model is evaluated using various performance measures routinely used in the literature. Two scenarios are evaluated, namely the proposed models without and with the existence of trust-related attacks.

Five metrics are used to measure the proposed model’s performance: accuracy, loss rate, precision, recall, and F-measure.

The level of agreement between an absolute and actual measurement is called accuracy. Accuracy is one of the most used classification performance measures, defined as the proportion of correctly classified samples to all samples [45] and computed using Equation (8).

(8) $$Accuracy=\frac{TP+TN}{TP+TN+FP+FN}$$

The loss rate is a function that, to facilitate learning, quantifies the difference between the training’s actual and projected output. Additionally, it helps to reduce error and evaluate the model performance [42]. The loss rate is calculated using Equation (9).

(9) $$Loss=-Y*Log\left(Y_{Pred}\right)-\left(1-Y\right)*\;Log\left(1-Y_{Pred}\right)$$

Precision describes a classification model’s capacity to select data points from a particular class. It is determined by dividing the quantity of correctly recovered samples by the total quantity of samples retrieved [46] and is given in Equation (10).

(10) $$Precision=\frac{TP}{TP+FP}$$

Recall is a classification model’s ability to identify each data point in a relevant class. It computes the ratio of the number of actual samples successfully retrieved to the total number of correct samples [47] and is defined in Equation (11).

(11) $$Recall=\frac{TP}{TP+FN}$$

Another measure, known as the *F-Measure*, which reflects the behavior of two measures, is obtained from precision and recall [38]. It is computed in Equation (12).

(12) $$F-Measure=2\cdot \frac{precision\;\cdot \;recall\;}{precision+recall}\;$$

### Trust-Related Attack Modeling Scenarios

Trust-related attacks are those committed by an untrusted entity, including offering an inadequate service or providing the trustor with unfavorable recommendations about the trustees [22,30]. Accordingly, this section’s purpose is to describe three scenarios of trust-related attacks.

**Figure 4:**
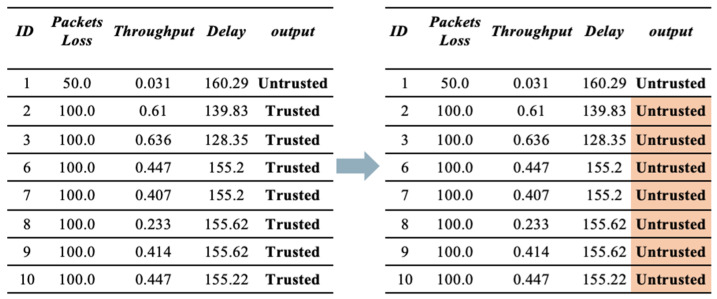
Sample of dataset before and after applying a bad-mouthing attack.

**(a)**  **First Scenario (Bad-mouthing Attack).** This attack aims to harm the reputation of good nodes to decrease their chances of being chosen as service providers [22]. Therefore, in this scenario, the reputation of trusted devices is manipulated to make them untrusted. As illustrated in Figure 4, the value of trust is manipulated by harming its reputation. Therefore, the devices will be identified as untrusted, despite them being trustworthy.

**Figure 5:**
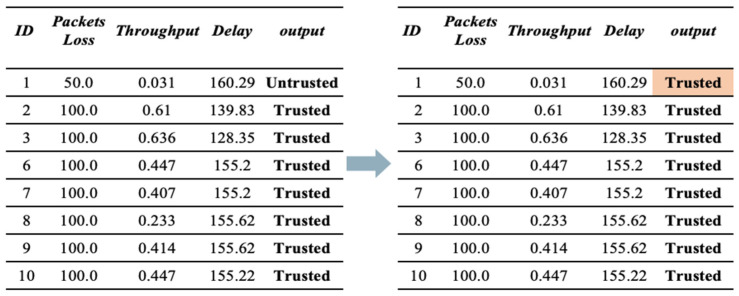
Sample of dataset before and after applying a good-mouthing attack.

**(b)**  **Second Scenario (Good-mouthing Attack).** This attack aims to improve the reputation of bad nodes to increase the probability of them being chosen as service providers [30]. Therefore, in this scenario, the reputation of untrusted devices is manipulated to make them trusted. As illustrated in Figure 5, the value of trust is manipulated by enhancing their reputation. Therefore, the devices will be identified as trusted, despite them being untrustworthy.

**Figure 6:**
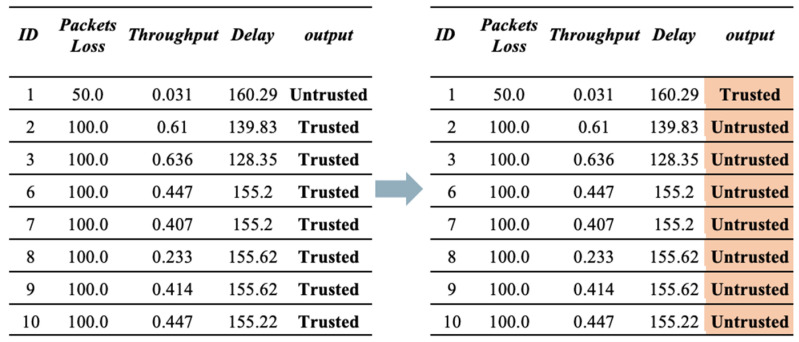
Sample of dataset before and after applying good-mouthing and bad-mouthing attacks.

**(c)**  **Third Scenario (Combination of both Scenario 1 and Scenario 2).** This scenario applies both bad-mouthing and good-mouthing attacks at the same time to the same dataset, as illustrated in Figure 6.

## 5. Experiments and Results

The experiment was conducted on Google CoLab with Python library packages such as Pandas, Numpy, Scikit-Learn, Matplotlib, and Keras [48]. Table 5 describes the details of the model setup.

The used dataset was split into training and test sets in a ratio of 70:30, respectively. To avoid overfitting and underfitting, the data were randomly divided several times until it was verified that the test set represented unseen behaviors.

### 5.1. Trust-Related Attack Modeling Scenarios

This subsection explores the results of the proposed model without and with trust-related attacks.

#### 5.1.1. Results of the Model without Trust-Related Attacks

**Figure 7:**
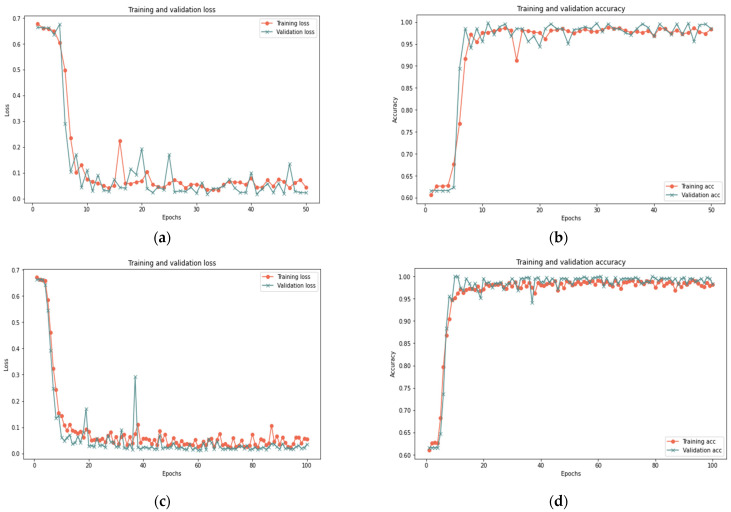
Results of the 25% sample size: (**a**) loss at 50 iterations; (**b**) accuracy at 50 iterations; (**c**) loss at 100 iterations; and (**d**) accuracy at 100 iterations.

**Figure 8:**
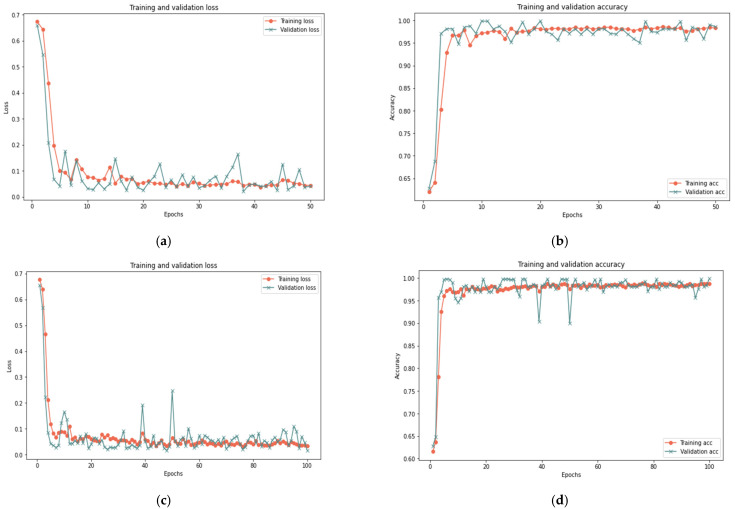
Results of the 50% sample size: (**a**) loss at 50 iterations; (**b**) accuracy at 50 iterations; (**c**) loss at 100 iterations; and (**d**) accuracy at 100 iterations.

**Figure 9:**
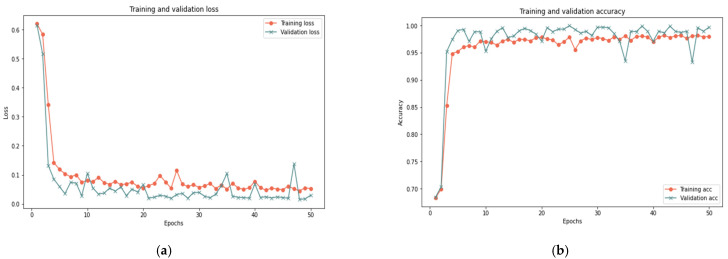

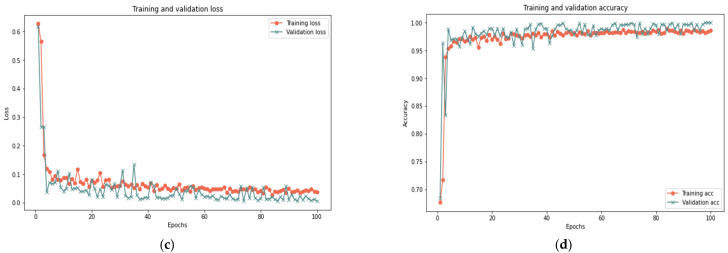
Results of the 100% sample size: (**a**) loss at 50 iterations; (**b**) accuracy at 50 iterations; (**c**) loss at 100 iterations; and (**d**) accuracy at 100 iterations.

This section analyzes the model using database samples of various sizes (25–100) and iterations (50 and 100) to assess the model’s efficacy under different dataset conditions. After 100 iterations, the model had reached 99.37% accuracy, 0.018 loss rate, 100% recall, 99.93% precision, and 99.65% F-measure in 420 s. Both 100 and 50 iterations provided comparable results for large samples of 25% and 50%, with a minor difference in the detection rate. Figure 7, Figure 8 and Figure 9 depict the accuracy and loss rate for each iteration with varying sample size, and Table 6 presents a detailed evaluation of the model.

Table 6 indicates that the proposed model successfully diagnosed behavior deviation using LSTM cells, yielding positive outcomes for various data sample sizes. Changing the sample size in the experiment revealed the suggested model’s adaptability to different circumstances. The minor variation in findings is normal and indicative of the proposed model’s capacity to handle small or large data samples. The model’s performance is improved with a more extensive test sample size. In addition, expanding the number of iterations has minimal effects on accuracy, recall, precision, and F-measure, but significant effects on time and loss rate, indicating that the model is refined with each iteration.

#### 5.1.2. Results of the Model with Trust-Related Attacks

In this section, the model has been tested on two datasets: the first is the normal dataset (the same dataset used in Section 5.1.1), and the other is the dataset after applying trust-related attacks (bad-mouthing and good-mouthing) with different numbers of iterations (50 and 100) and different sizes of dataset (25%, 50%, and 100%) to prove the effectiveness of the proposed model in identifying such attacks.

##### First Scenario Results

**Figure 10:**
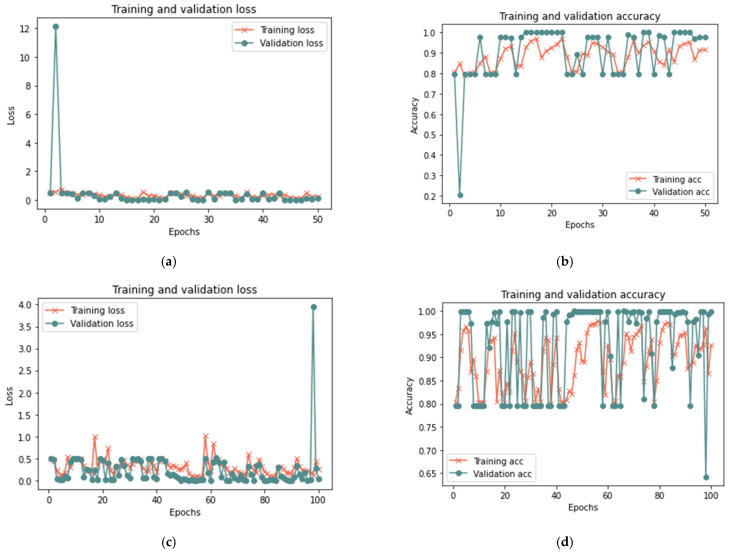
Results of the first scenario with 25% sample size: (**a**) loss at 50 iterations; (**b**) accuracy at 50 iterations; (**c**) loss at 100 iterations; and (**d**) accuracy at 100 iterations.

**Figure 11:**
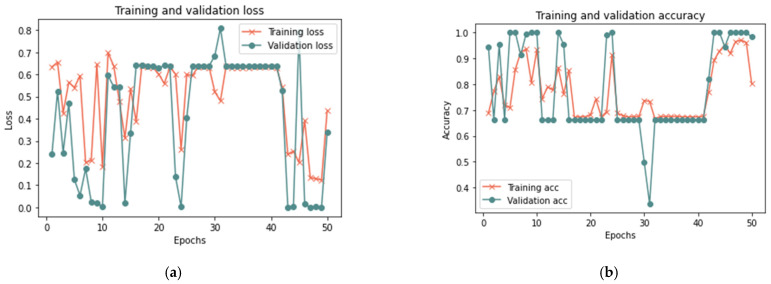

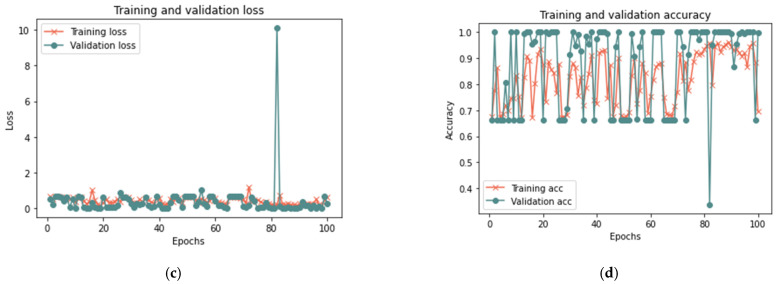
Results of the first scenario with 50% sample size: (**a**) loss at 50 iterations; (**b**) accuracy at 50 iterations; (**c**) loss at 100 iterations; and (**d**) accuracy at 100 iterations.

**Figure 12:**
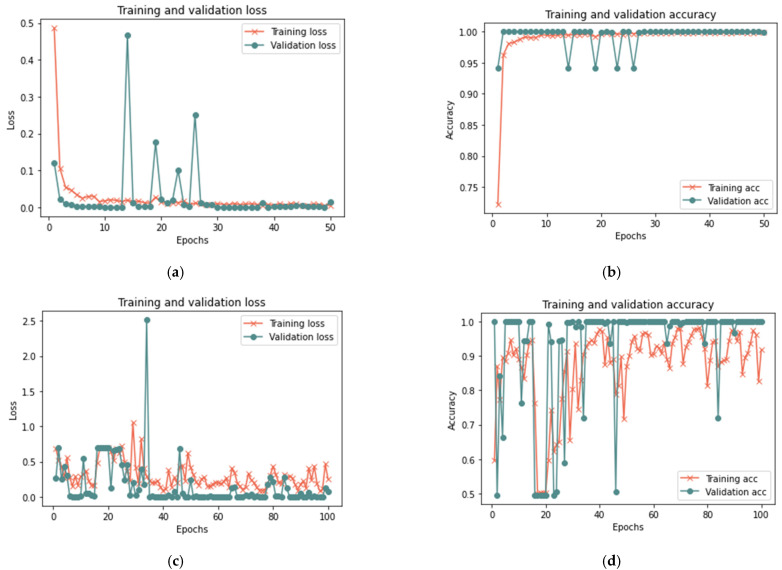
Results of the first scenario with 100% sample size: (**a**) loss at 50 iterations; (**b**) accuracy at 50 iterations; (**c**) loss at 100 iterations; and (**d**) accuracy at 100 iterations.

Table 7 indicates that the suggested model for a sample size of 100% yields positive results after 50 iterations, achieving 99.96% accuracy, 0.0130 loss rate, 99.92% recall, 100% precision, and an F-measure of 99.96%, in 15 min. After 20 min and 100 iterations, the model achieved 99.98% accuracy, 0.0723 loss rate, 99.98% recall, 100 percent precision, and 99.98% F-measure. Similar findings are reported for 100 and 50 iterations for sample sizes of 25% and 50%, with a minor change in the detection time. Figure 10, Figure 11 and Figure 12 depict the accuracy and the loss rate for each iteration with different sample sizes for this scenario.

According to the results shown in Table 7, it is clear that the proposed model can detect the manipulations that occurred in the trust value due to a bad-mouthing attack. Compared to the proposed model without an attack, the results demonstrate an increased detection time with an attack. Hence, the detection time ranged from 5 min to 20 min for all sizes of dataset used, whereas the model without attack had a detection time ranging from less than 1 min to 2 min. With the presence of a bad-mouthing attack, the results showed an increase in detection effectiveness with the expansion in the data size used. With increasing sample size, the performance metrics such as recall, precision, and the F-1 measure increase and the loss rate decreases.

##### Second Scenario Results

**Figure 13:**
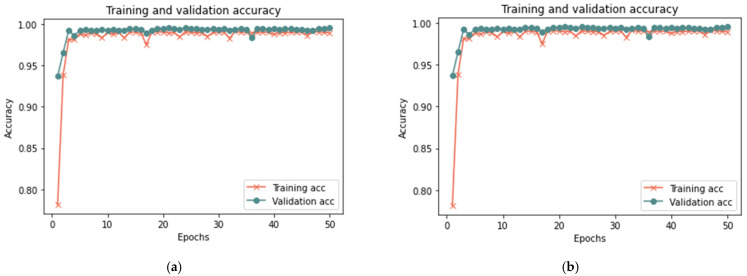

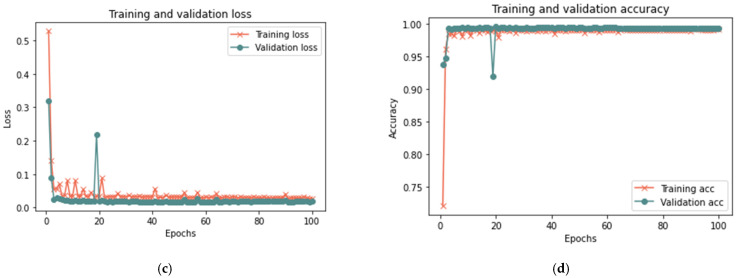
Results of the second scenario with a 25% sample size: (**a**) loss at 50 iterations; (**b**) accuracy at 50 iterations; (**c**) loss at 100 iterations; and (**d**) accuracy at 100 iterations.

**Figure 14:**
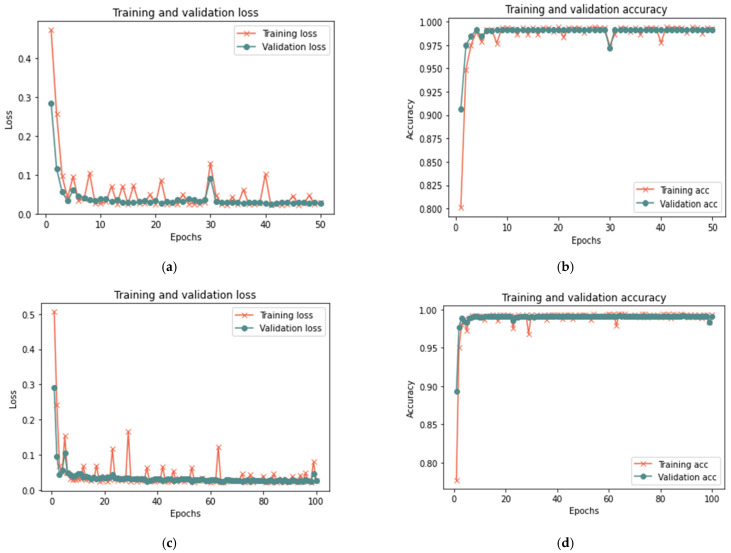
Results of the second scenario with 50% sample size: (**a**) loss at 50 iterations; (**b**) accuracy at 50 iterations; (**c**) loss at 100 iterations; and (**d**) accuracy at 100 iterations.

**Figure 15:**
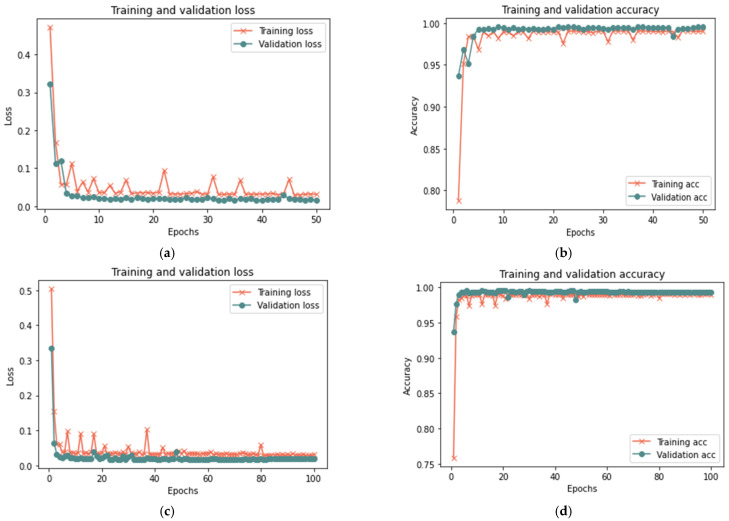
Results of the second scenario with 100% sample size: (**a**) loss at 50 iterations; (**b**) accuracy at 50 iterations; (**c**) loss at 100 iterations; and (**d**) accuracy at 100 iterations.

Table 8 shows that the proposed model for the total sample size exhibited better results in 50 iterations, as it achieved 99.96% accuracy, 0.0174 loss rate, 99.92% recall, 100% precision, and 99.96% F-measure in 18 min. After 100 iterations, the model shows some improvement, having achieved 99.98% accuracy, 0.0182 loss rate, 99.98% recall, 100% precision, and 99.98% F-measure in 20 min. Figure 13, Figure 14 and Figure 15 illustrate each iteration’s accuracy and loss rate with varying sample sizes.

Based on the results reported in Table 9 and Figure 13, Figure 14 and Figure 15, it was evident that the LSTM method employed in the suggested model could spot changes in the trust value as a result of a good-mouthing attack. Compared to the models without an attack, the findings demonstrated a longer detection time in the presence of an attack. As a result, the model without attack had a detection time of less than 1 to 2 min, but the detection time for all sizes of dataset used in this phase varied from 5 to 20 min. The findings showed the efficacy of detecting a good-mouthing attack as the volume of data utilized grew. In contrast, the loss rate declined as the sample size increased, whereas accuracy, recall, precision, and F-measure all increased.

##### Third Scenario Results

**Figure 16:**
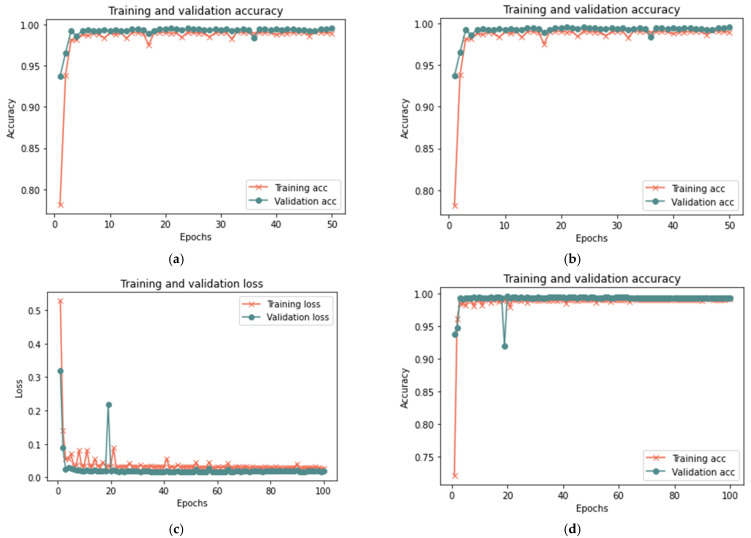
Results of the third scenario with 25% sample size: (**a**) loss at 50 iterations; (**b**) accuracy at 50 iterations; (**c**) loss at 100 iterations; and (**d**) accuracy at 100 iterations.

**Figure 17:**
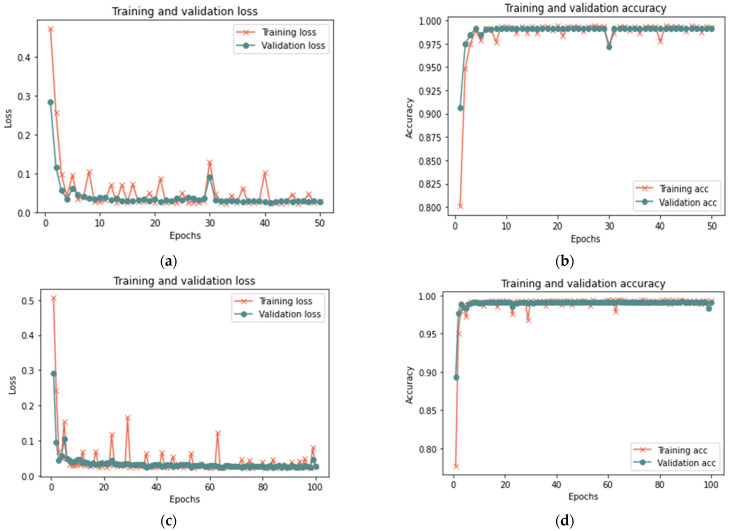
Results of the third scenario with 50% sample size: (**a**) loss at 50 iterations; (**b**) accuracy at 50 iterations; (**c**) loss at 100 iterations; and (**d**) accuracy at 100 iterations.

**Figure 18:**
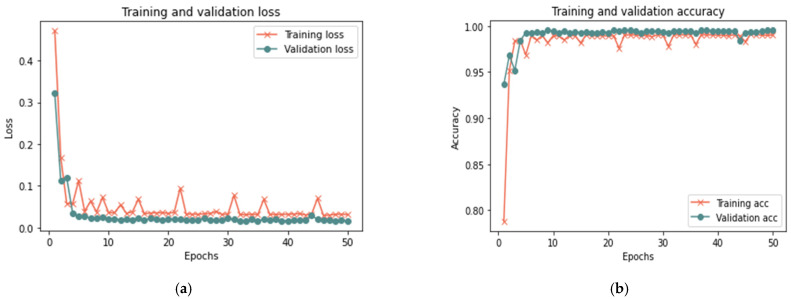

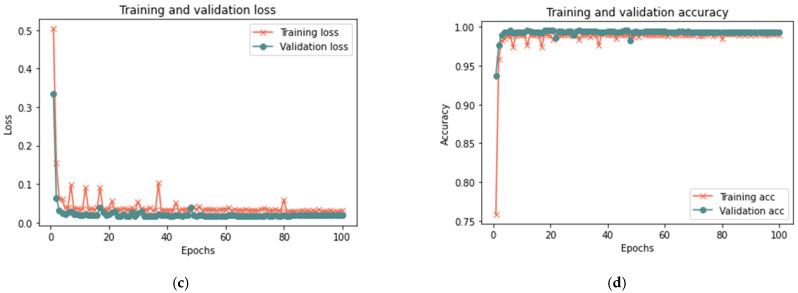
Results of the third scenario with 100% sample size: (**a**) loss at 50 iterations; (**b**) accuracy at 50 iterations; (**c**) loss at 100 iterations; and (**d**) accuracy at 100 iterations.

Table 9 shows that the proposed model for the total sample size exhibited better results in 50 iterations, as it achieved 99.33% accuracy, 0.0182 loss rate, 98.67% recall, 100% precision, and 99.96% F-measure in 18 min. After 100 iterations, the model shows some improvement, since it achieved 99.28% accuracy, 0.0182 loss rate, 98.59% recall, 100% precision, and 99.29% F-measure in 20 min. Figure 16, Figure 17 and Figure 18 illustrate each iteration’s accuracy and loss rate with varying sample size.

Based on the results reported in Table 9 and Figure 16, Figure 17 and Figure 18, the model was able to identify untrusted entities with high effectiveness with an increase in time. The outcomes demonstrated a clear correlation between accuracy, recall, and F-measure. In addition, the number of iterations and loss rate are inversely related. The proposed model in the presence of more than one attack required a longer detection time than the model without attacks or in the presence of one attack, as the time ranged from 15 to 20 in the presence of more than one type of attack. The results show that the proposed model can detect untrusted entities during trust-related attacks.

In conclusion, the results of the proposed model revealed that the LSTM technique could deal with different data sizes and more effectively learn complex the behavioral patterns of IoT devices. As a result, the proposed model can improve the reliability of IoT services and devices while also adapting to complex and unknown trust-related behaviors.

#### 5.1.3. Comparison with Existing Deep Learning Models

Deep learning models have been adapted to the field of detection. Three deep learning architectures have been used in the literature (LSTM, MLP, and ANN) to detect untrusted entities in IoT devices and services. It is important to consider the nature of the data that will be tested in order to achieve the goal for which the model was designed. Despite the fact that deep learning is frequently employed to handle issues with vast amounts of data and continuous changes in behavior, each model is developed with a particular goal in mind depending on the dataset.

Comparing the proposed model with other models that have been used in related works reveals that LSTM is frequently utilized for complex learning tasks, such as prediction tasks, spotting behavioral changes [41], machine translation [49], and handwriting creation [50], whereas ANN is frequently used for image processing, character recognition, and forecasting and MLP is typically employed for image processing tasks [51]. The data used in this study come from IoT device actions, hence it is they are behavioral pattern data. As result, the proposed model is more suitable for identifying changes in trustworthy and untrusted behaviors; consequently, it can counter trust-related attacks.

## 6. Conclusions

Managing trust is an issue with far-reaching consequences for artificial societies, such as those with IoT devices. Increased dependence on IoT devices and services has aggravated this issue recently. Existing models are no longer effective in the age of big data and with IoT devices with a dynamic nature and heterogeneity. The proposed research in this paper suggested a model for trust management in IoT devices and services based on the LSTM technique. The proposed model aimed to identify untrusted entities in IoT services and isolate untrusted devices. The effectiveness of the proposed model was evaluated using different data samples with different sizes. The LSTM technique was used to detect changes in behavior with high accuracy. The experimental results demonstrated the model’s ability to recognize untrusted entities when there are trust-related attacks. As a result, the suggested model can improve IoT device and service reliability and adapt to complicated and unknowable behaviors. Future work will consider more features to calculate the trust value (e.g., energy consumption). In addition, more trust-related attack scenarios will be considered (e.g., on–off and discriminatory attacks).

## Figures and Tables

**Table 1:** Trust-related attacks.

Attack Name	Discretion
Good-mouthing	A node will enhance its reputation to be selected as a service provider [21].
Bad-mouthing	An attack in which malicious nodes attempt to ruin well-behaved nodes’ reputations to minimize their probability of being chosen as service providers [22].
Ballot-stuffing	An attack in which malicious nodes attempt to enhance other malicious nodes’ reputations to increase their probability of being chosen as providers of services [22].
On–off	An attacker can attempt to disturb a trust redemption scheme in this form of attack by both behaving well and badly; therefore, trust is always redeemed just before another attack occurs [23].
Discriminatory	An attack in which malicious nodes, based on human inclinations to strangers, discriminatorily attack other nodes against a clear social relationship [22].

**Table 2:** Summary of existing studies.

Reference	Not Dealing with Trust-Related Attacks	Dealing with a Single Type of Trust-Related Attack	Dealing with Multiple Types of Trust-Related Attacks
[24]	√		
[25]	√		
[10]		√	
[26]	√		
[11]		√	
[13]	√		
[27]	√		
[6]	√		
[28]			√
[29]	√		
[12]	√		
[20]	√		
[30]	√		
[31]	√		
[32]	√		
[33]	√		
[34]	√		
[35]	√		

**Table 5:** Model setup.

Parameter	Value
Language	Python
Libraries	Pandas, Numpy, Scikitlearn, Matplotlib, and Keras
Training Set	70%
Test Set	30%
Input Layer	1
LSTM Cells	Two cells
Activation Functions	Rectified Linear Unit (ReLu), and sigmoid
Dense Layer	1
Dropout	0.20
Optimizer	Adam
Number of Epochs	50 and 100

**Table 6:** Experimental results.

Sample Size = 25%						
Iterations	Accuracy (%)	Loss Rate	Recall (%)	Precision (%)	F-Measure (%)	Time (s)
50	98.33	0.0350	99.85	95.97	97.87	88
100	98.50	0.00223	96.70	99.38	98.02	180
**Sample Size = 50%**						
**Iterations**	**Accuracy (%)**	**Loss Rate**	**Recall (%)**	**Precision (%)**	**F-Measure (%)**	**Time (s)**
50	98.62	0.0125	99.92	96.50	98.18	84
100	99.81	0.0115	99.92	99.69	99.81	185
**Sample Size = 100%**						
**Iterations**	**Accuracy (%)**	**Loss Rate**	**Recall (%)**	**Precision (%)**	**F-Measure (%)**	**Time (s)**
50	99.66	0.0082	98.986	100	99.49	356
100	99.97	0.0059	100	99.92	99.96	420

**Table 7:** Experimental results of the first scenario.

Sample Size = 25%						
Iterations	Accuracy (%)	Loss Rate	Recall (%)	Precision (%)	F-Measure (%)	Time (s)
50	97.69	0.088	88.70	100	94.01	5 m
100	99.93	0.029	100	99.69	99.85	8 m
**Sample Size = 50%**						
**Iterations**	**Accuracy (%)**	**Loss Rate**	**Recall (%)**	**Precision (%)**	**F-Measure (%)**	**Time (s)**
50	98.25	0.033	94.80	100	97.33	10 m
100	99.73	0.0255	99.21	100	99.60	12 m
**Sample Size = 100%**						
**Iterations**	**Accuracy (%)**	**Loss Rate**	**Recall (%)**	**Precision (%)**	**F-Measure (%)**	**Time (s)**
50	99.96	0.0130	99.92	100	99.96	15 m
100	99.98	0.0723	99.98	100	99.98	20 m

**Table 8:** Experimental Results of the second scenario.

Sample Size = 25%						
Iterations	Accuracy (%)	Loss Rate	Recall (%)	Precision (%)	F-Measure (%)	Time (s)
50	98.51	0.0188	88.70	100	94.01	6 m
100	99.93	0.0190	100	99.69	99.85	8 m
**Sample Size = 50%**						
**Iterations**	**Accuracy (%)**	**Loss Rate**	**Recall (%)**	**Precision (%)**	**F-Measure (%)**	**Time (s)**
50	99.25	0.027	94.80	100	97.33	10 m
100	99.73	0.017	99.21	100	99.60	12 m
**Sample Size = 100%**						
**Iterations**	**Accuracy (%)**	**Loss Rate**	**Recall (%)**	**Precision (%)**	**F-Measure (%)**	**Time (s)**
50	99.96	0.0174	99.92	100	99.96	18 m
100	99.98	0.0182	99.98	100	99.98	20 m

**Table 9:** Experimental Results of the third scenario.

Sample Size = 25%						
Iterations	Accuracy (%)	Loss Rate	Recall (%)	Precision (%)	F-Measure (%)	Time (s)
50	99.53	0.0180	99.10	99.96	99.53	10 m
100	99.33	0.0180	98.67	100	99.33	15 m
**Sample Size = 50%**						
**Iterations**	**Accuracy (%)**	**Loss Rate**	**Recall (%)**	**Precision (%)**	**F-Measure (%)**	**Time (s)**
50	99.13	0.027	97.26	100	98.61	14 m
100	99.13	0.017	97.26	100	98.61	16 m
**Sample Size = 100%**						
**Iterations**	**Accuracy (%)**	**Loss Rate**	**Recall (%)**	**Precision (%)**	**F-Measure (%)**	**Time (s)**
50	99.33	0.0184	98.67	100	99.33	17 m
100	99.28	0.0182	98.59	100	99.29	20 m

## Data Availability

Not applicable.
